# Toolbox for Non-Intrusive Structural and Functional Analysis of Recombinant VLP Based Vaccines: A Case Study with Hepatitis B Vaccine

**DOI:** 10.1371/journal.pone.0033235

**Published:** 2012-04-06

**Authors:** Anke M. Mulder, Bridget Carragher, Victoria Towne, Yuan Meng, Yang Wang, Lance Dieter, Clinton S. Potter, Michael W. Washabaugh, Robert D. Sitrin, Qinjian Zhao

**Affiliations:** 1 Vaccine Analytical R&D, Merck Research Laboratories, West Point, Pennsylvania, United States of America; 2 Vaccine Bioprocess R&D, Merck Research Laboratories, West Point, Pennsylvania, United States of America; 3 Merck Manufacturing Division, Vaccine Manufacturing Science and Commercialization, West Point, Pennsylvania, United States of America; 4 Department of Research & Development, NanoImaging Services, La Jolla, California, United States of America; Faculty of Biochemistry Biophysics and Biotechnology, Jagiellonian University, Poland

## Abstract

**Background:**

Fundamental to vaccine development, manufacturing consistency, and product stability is an understanding of the vaccine structure-activity relationship. With the virus-like particle (VLP) approach for recombinant vaccines gaining popularity, there is growing demand for tools that define their key characteristics. We assessed a suite of non-intrusive VLP epitope structure and function characterization tools by application to the Hepatitis B surface antigen (rHBsAg) VLP-based vaccine.

**Methodology:**

The epitope-specific immune reactivity of rHBsAg epitopes to a given monoclonal antibody was monitored by surface plasmon resonance (SPR) and quantitatively analyzed on rHBsAg VLPs in-solution or bound to adjuvant with a competitive enzyme-linked immunosorbent assay (ELISA). The structure of recombinant rHBsAg particles was examined by cryo transmission electron microscopy (cryoTEM) and in-solution atomic force microscopy (AFM).

**Principal Findings:**

SPR and competitive ELISA determined relative antigenicity in solution, in real time, with rapid turn-around, and without the need of dissolving the particulate aluminum based adjuvant. These methods demonstrated the nature of the clinically relevant epitopes of HBsAg as being responsive to heat and/or redox treatment. In-solution AFM and cryoTEM determined vaccine particle size distribution, shape, and morphology. Redox-treated rHBsAg enabled 3D reconstruction from CryoTEM images – confirming the previously proposed octahedral structure and the established lipid-to-protein ratio of HBsAg particles. Results from these non-intrusive biophysical and immunochemical analyses coalesced into a comprehensive understanding of rHBsAg vaccine epitope structure and function that was important for assuring the desired epitope formation, determinants for vaccine potency, and particle stability during vaccine design, development, and manufacturing.

**Significance:**

Together, the methods presented here comprise a novel suite of non-intrusive VLP structural and functional characterization tools for recombinant vaccines. Key VLP structural features were defined and epitope-specific antigenicity was quantified while preserving epitope integrity and particle morphology. These tools should facilitate the development of other VLP-based vaccines.

## Introduction

Recombinant virus-like particles (VLPs) are attractive candidates for vaccine design because, while they resemble native virions in size and morphology, they are non-infectious due to the absence of a viral genome. VLP surface structure can be engineered and optimized to achieve an enhanced immune response due to the multiplicity of repetitive surface epitopes that mimic the authentic surface features of native virions [1]. Since its initial introduction (Table S1), the VLP approach has been extended to include production in a variety of cell culture systems; recombinant technology has successfully been used to produce VLPs as vaccines [2], [3] and as epitope presentation vehicles for HIV (gp41) [4] and malaria [5].

Non-infectious VLPs derived from Hepatitis B virus (HBV) and composed of the small HBV derived surface antigen (HBsAg) were described over 40 years ago [6], [7]. HBsAg VLPs are 22 nm spherical lipid-protein particles [6], [7] composed of three envelope proteins [8], that produce a strong protective immune response [9]. The envelope proteins result from variations in post translational proteolytic processing of the HBV S gene protein product and are termed the small (S), medium (M), and large (L) form of HBsAg, or the S protein [10]. Non-infectious VLPs self-assembled from these proteins and lipid were used for the development of the first VLP-based vaccine and, a few years later, recombinant DNA technology was employed to make the first recombinant protein based vaccine with only the S form of HBsAg (226 aa) [11].

HBsAg VLPs induce a protective immune response by mimicking the surface features of native HBV virions by including multiple and repetitive epitopes. Well-defined epitope structure and less protein conformation flexibility are important for binding to neutralization antibodies *in vitro* (antigenicity) and in eliciting neutralizing antibodies *in vivo* (immunogenicity) for the vaccine particles [12], [13]. The immunogenic epitope of HBV recognized by neutralizing antibody RF1 is composed of a stretch of 14 amino acid residues, 8 of which are cysteines. Refinement of epitope structure, a process known as maturation via disulfide bond formation and exchange at these cysteine residues during purification or storage over time [1], [14], [15], [16], [17], has been previously linked to subviral particle antigenic and immunogenic properties [12], [14]. In order to ensure recombinant vaccine safety and efficacy, it is vital to facilitate the development and maintenance of these native virion-like epitopes on the VLP surface [3], [18] in a manner that enhances VLP stability during protein expression, purification, and formulation. This requires an in-depth understanding of VLP epitope structure and function.

Although hundreds of millions of patients have been immunized by the recombinant HBsAg VLP based HBV vaccines since the initial introduction for human use in 1986, a definitive understanding of the HBsAg VLP structure and the key antigenic features is still lacking. Characterization efforts have been hampered by the conformational heterogeneity inherent in a lipid-protein particle with 75% protein and ∼25% lipid, the important role of disulfide bond formation for epitope structure and function of this cysteine-rich VLP, and the presence of the S, M, and L forms of the HBsAg protein in early preparations [19], [20], [21]. In addition, traditional structural and antigenicity characterization methods are prone to artifacts resulting from sample processing steps (Table 1).

**Table 1:** Non-intrusive biophysical and immunochemical methods evaluated here for VLP characterization compared with conventional techniques.

	Conventional Method	Non-intrusive Toolbox
**Transmission Electron Microscopy (TEM)**	**TEM with Negative Stain Sample Preparation**	**Cryo-TEM with Vitrified Sample Preparation**
	- Adsorption to surface of grid can cause particle deformation and aggregation	- No adsorption to surface
	- Washing steps can change sample buffer conditions	- No washing steps
	- Heavy metal stain can cause particle deformation and dehydration	- No heavy metal stain; Sample is preserved in formulation buffer in frozen-hydrated state
	*Potential for changes in epitope structure during sample processing*	*Epitope structures are preserved during sample processing*
**Atomic Force Microscopy (AFM)**	**AFM on Mica Surface with Dried Samples**	**AFM in Solution on Mica Surface with Flow Cell**
	- Adsorption to surface of mica can cause particle deformation, aggregation, and dehydration	- No adsorption to surface
	- Washing steps can change sample buffer conditions	- No washing steps
	- Scanning mode can cause particle deformation	- Flow cell analyzes sample in solution; Tapping mode is less likely to cause particle deformation
	*Potential for changes in epitope structure during sample processing*	*Epitope structures are preserved during sample processing*
**Antibody Binding or Antigenicity Analysis**	**Radio-labeled Immuno Assay (RIA) or Enzyme-linked Immunosorbent Assay (ELISA)**	**Surface Plasmon Resonance (SPR)**
	- Sample binding is analyzed adsorbed to a surface	- Sample binding is analyzed in solution
	- Radio or enzyme label required for binding signal amplification	- No label required for binding signal amplification; Signal is directly proportion to mass deposited on surface; Assay can be used for real-time kinetic monitoring or end-point binding analysis [23]
	- Turn-around time is 0.5-3 days	- Turn-around time is 20-30 minutes
	*Indirect link between signal and binding*	*Direct link between signal and binding*
**ELISA for Antigenicity Determination for Vaccines**	**Sandwich ELISA**	**Solution Competitive ELISA** *
	- Adsorption to surface can cause particle deformation and dehydration	- No adsorption to surface
	- Washing steps can change sample buffer conditions	- No washing steps; Sample is analyzed in solution
	- Dissolution of sample from particulate adjuvant required for analysis	- Sample can be analyzed upon adsorption to adjuvant
	*Potential for changes in epitope structure during sample processing*	*Epitope structures are preserved during sample processing*

* The solution competitive ELISA measures the accessible epitopes on VLPs adsorbed to particulate adjuvant. This method is used to probe the stability samples upon prolonged storage in a more faithful manner as to the intact and accessible epitopes. In addition, this method may also mimic the *in vivo* antigen presentation to some degree without the needs to dissolve the aluminum adjuvant, which is co-injected with antigen during immunization.

Structural methods, such as negative stain transmission electron microscopy (TEM) or atomic force microscopy (AFM) on surface immobilized VLPs, require sample adsorption to a surface, washing steps, and additives that can result in particle dehydration, aggregation, and deformation. More widely accepted antigenicity characterization methods, such as animal based mouse potency assays, radio-labeled immuno assays (RIA), and enzyme-linked immunosorbent assays (ELISA), suffer from an indirect correlation between signal and antigen content due to requirements for secondary labels, signal amplification, or presence of multiple epitopes [12], [13]. Furthermore, these methods also require washing steps, sample adsorption to surfaces, and adjuvant dissolution, which can lead to sample dehydration and deformation. The potential for artifact introduction during sample processing by these traditional structural and functional characterization tools complicate data interpretation, as there is not a true correlation between native epitope structure and measurement.

Recent developments in non-intrusive biophysical and immunochemical methods that allow analysis of samples in their native state provide the potential for direct measurement and understanding of epitope structure and function [14], [22], [23], [24], [25], [26]. Surface plasmon resonance (SPR) directly and rapidly monitors antigenicity development during epitope refinement in real time without the requirement of a label, where the signal is proportional to the mass deposited onto the sensor chip surface during biomolecular interactions [23]. An optimized solution competitive ELISA, performed with a neutralizing monoclonal antibody (mAb) that targets clinically relevant epitopes [23], quantitatively tracks the development of virion-like epitopes on VLPs in solution or when bound to particulate aluminum-based adjuvant. CryoTEM allows determination of the native, hydrated structure of VLPs [24], [25], and in-solution AFM visualizes VLP surface features without particle deformation or drying [14], [22], [26]. Here, we have evaluated the use of these biophysical and immunochemical methods in concert as a potential novel suite of non-intrusive VLP characterization tools by applying them to the characterization of the VLP-based vaccine against HBV [11].

## Results

### Functional Activity of rHBsAg Epitopes with SPR and ELISA

Since mouse potency tests and in vitro relative potency analyses reflect the overall immune response in animals or multiple epitopes on antigen, probing the antigenicity of vaccine preparations and process intermediates, and the immune response with respect to a given epitope or a set of epitopes helps to assess the epitope-specific antigenicity or the functionality of the binding antibody titers. RF1 and A1.2 are two protective murine monoclonal antibodies (mAbs) known to recognize the immune dominant region on the surface loop of HBsAg near the dimer interface and have high degree of sensitivity to VLP surface and epitope conformation [12], [23], [27], [28], [29], [30]. Therefore, a panel of mAbs, with these two mAbs included, was used to analyze the rHBsAg particle in solution over the course of bioprocessing and the final product bound to adjuvant by competitive ELISA and SPR.

**Figure 1:**
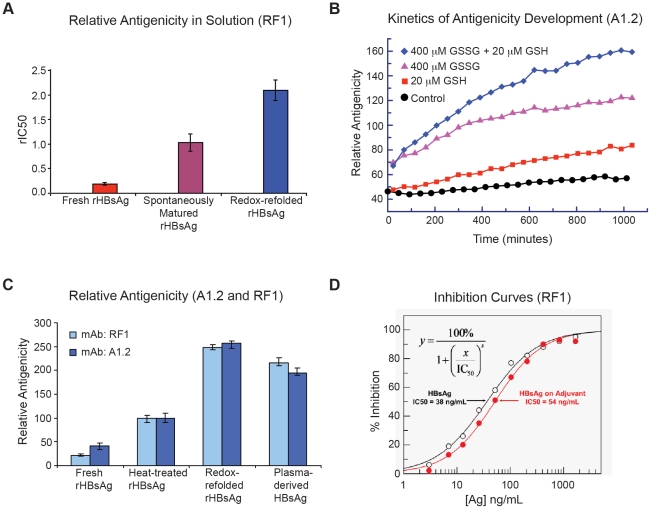
Measurement of rHBsAg VLP antigenicity by SPR or solution competitive ELISA. (A) Solution competition ELISA measurement (or relative IC50s) for freshly prepared (n = 5), heat-treated (n = 5), and redox-refolded (n = 3) rHBsAg VLPs; (B) A1.2 epitope maturation over time in PBS and with a redox buffer-reduced (GSH) and/or oxidized (GSSG) forms of glutathione; (C) relative antigenicity by SPR with A1.2 and RF1 (n = 12); (D) Relative antigenicity of rHBsAg in aqueous solution as well as when adsorbed onto particulate adjuvant.

The solution competition sandwich ELISA experiment revealed increased antigenicity for spontaneously matured and redox-refolded particles (Figure 1A), and for particles further along in bioprocessing (Figure S1A). Different mAbs displayed different levels of sensitivity for the antigen and varying levels of end point antigenicity, with RF1 showing among the highest antigenicity (Figure S1B). RF1 is the only mouse IgG that has been demonstrated to have protective activity in non-human primates against viral challenge [27], [28], [31]. This mAb binds the HBsAG epitope at an intramolecularly disulfide-bonded cyclic peptide important for viral entry [27], [28], [31].

The SPR setup allowed quantitative monitoring of VLP maturation over time and supported results obtained by the ELISA platform that antigenicity is increased for HBsAg VLPs with higher disulfide content. Increased antigenicity was observed over the course of maturation and for redox-treated rHBsAg VLPS (Figure 1B). Marked increase in antigenicity was observed for heat- and redox-treated rHBsAg VLPs (Figure 1C). The latter were over 10-fold more antigenic than freshly purified VLPs and had comparable antigenicity to plasma-derived particles (Figure 1C). Together with our ELISA study, these results support the notion that disulfide bond formation and/or isomerization, which occurs during VLP maturation in bioprocessing, induces changes in epitope structure that are vital for optimal antigenicity [12], [14], [23], [28].

Almost all HBsAg based vaccines are administered with aluminum adjuvants, thus understanding the available epitopes on the adjuvanted VLPs is critical as it mimics to some degree the antigen presentation after intra muscular injection. Lot-to-lot comparison of inhibition curves obtained for antigen in solution with antigen bound to adjuvant showed similar IC50 values when monitored with RF1 (Figure 1D and Table S2). This indicates that antigenicity was not adversely affected by adsorption to adjuvant.

SPR and competitive ELISA, with conformation-sensitive and/or disulfide-dependent mAbs allowed quantitative determination of epitope development during process manufacturing and in final vaccine products by monitoring relative antigenicity in solution, in real time, and with rapid turn-around. Utilization of multiple mAbs in conjunction with a quantitative assay that could be performed for the antigen in solution and bound to adjuvant assured more complete understanding of the VLP key epitope make up and correlation to vaccine immunogenicity. These two quantitative antigenicity analysis methods are complementary to the commonly used sandwich ELISA and mouse potency assays for vaccine batch release and stability testing [12], [13]. Similar application of the epitope-specific antigenicity analysis using competitive ELISA (IC50) was recently reported for another recombinant based VLP, human papillomavirus (HPV) type 16, using a panel of mAbs to delineate the specific changes in mAb binding pattern with or without VLP disassembly/reassembly [32].

### VLP Morphology by In-Solution AFM and CryoTEM

**Figure 2:**
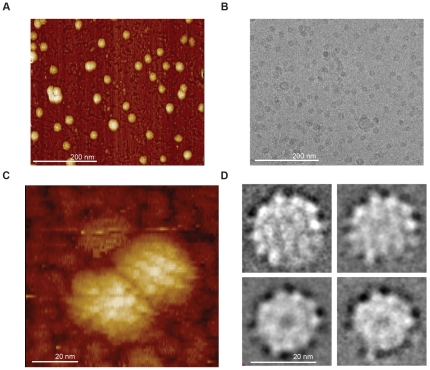
AFM and cryoTEM analysis of rHBsAg particles. (A) AFM field of view, (B) cryoTEM field view, (C) high-magnification AFM view, and (D) two-dimensional CryoTEM class averages of rHBsAg VLP particles. Note that image contrast is reversed in (B) and (D).

The conformational heterogeneity [19], [20], [21] of HBsAg particles has long hampered a definitive structural understanding of the key antigenic features of this cysteine- and proline-rich VLP (Table S3). Here we analyzed the size, shape, and morphology of a conformationally homogenous, redox-treated form of yeast derived HBsAg vaccine particles [23] (rHBsAg) using cryoTEM and in-solution AFM. These particles were assembled from only the active S-protein component of the HBV vaccine. Visual inspection of AFM and cryoTEM images revealed mostly spherical rHbsAg VLPs with slightly amorphous boundaries (Figure 2A–B). Higher magnification AFM views of the particles showed slight protrusions from the VLP surface in a regular pattern (Figure 2C) that was in agreement with the pattern of protrusions observed in 2D class averages obtained from cryoTEM data (Figure 2D). Size analysis of spherical VLPs by in-solution AFM measurements revealed an average particle size of ∼22 nm in agreement with previous studies [26], [27] (Figure S2B). Quantitative particle size distribution analysis of 266 particles in cryoTEM images revealed a maximum particle Feret diameter ranging 18.5–22.7 nm and a circularity measure of 0.89+/−0.03, indicating that the VLP morphology deviates from perfect circularity (Figure S2A).

VLP morphology was further investigated before and after oxidative treatment. In-solution AFM revealed that, after oxidative treatment, mature rHBsAg VLPs have better-defined surface protrusions and a lesser degree of particle flattening (Figures S3). Similarly, cryoTEM images showed more compact VLP particles with better-defined borders in non-reducing environments (Figure S4). These results combined with our SPR and ELISA results support previous studies [12], [14], [23], and suggest a fundamental link between antigenicity, disulfide bond formation, and epitope structure.

### VLP Structural Characterization by CryoTEM 3D Reconstruction

**Figure 3:**
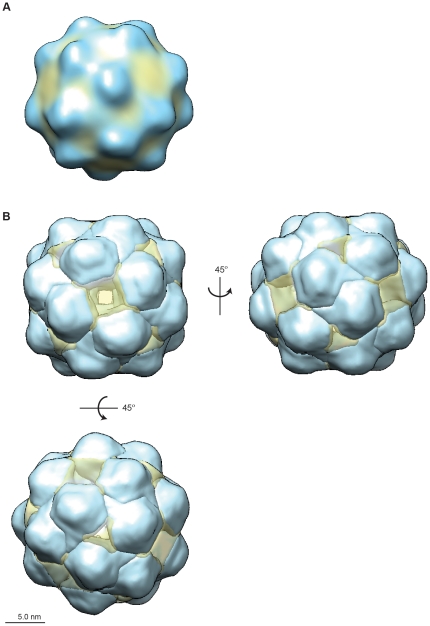
CryoTEM map of rHBsAg lipid-protein particle. (A) The resulting 3D map presented with roughly spherical morphology with “knuckle”-like protrusions projecting from a smooth surface. (B) Segmentation of the map revealed regions of high density, presumed to be protein, surrounded by regions of lesser density, presumed to be lipid. Map shown end-on for the 4-fold (top left), 2-fold (top right), and 3-fold (bottom left) views.

Only spherical, single rHBsAg VLPs were selected from 211 cryoTEM images and subjected to 2D alignment and classification in preparation for 3D reconstruction. The resulting class averages revealed two predominant particle populations, measuring ∼20 nm and ∼22 nm in diameter, respectively (Figure S5). Each size population was subjected to 3D reconstruction individually and refined to a similar 3D structure (Figure S6); as such, particle size populations were combined and subjected to 3D refinement with octahedral symmetry [19], [33]. The resulting map presented with roughly spherical morphology and “knuckle”-like protrusions projecting from a smooth surface (Figure 3A).

The basic structural morphology was in agreement with that obtained for human plasma-derived particles [12], [23], which are comparable to the redox-refolded rHBsAg VLPs used in our study. It is possible that the variety of proposed structures (Table S3) may be the result of heterogeneity, not only in the S, M, and L forms of HBsAg for plasma-derived particles or tubules, but also in degree of cross-linking and proper disulfide bond pairings, which are vital for retention of an ordered structure with key antigenic features [14], [34]. Segmentation of the cryoTEM map into discrete regions of density resulted in 24 “knuckle”-like protrusions, presumed to be S-protein, separated by regions of lesser density, presumed to be lipid (Figure 3B). In accordance with octahedral symmetry, the rHBsAg particle showed rotational axes of 4-, 3-, and 2-fold symmetry, obtained by rotating the particle by 45° (Figure 3B). A cross section of the map revealed an empty internal core surrounded by a tightly packed S-protein frame with lipids interspersed (Figure S7).

**Figure 4:**
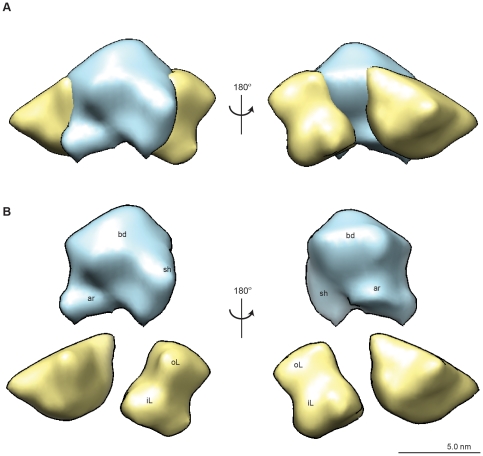
Structural features of the protein containing protrusion and surrounding lipid layer. (A) A single protein protrusion with associated lipid was segmented from the 3D map and (B) various structural features present in the lipid and protein densities were identified. The protein unit is composed of a body (bd), shoulder (sh), and arm (ar) region, and the lipid layer is composed of an outer layer (oL) and an inner layer (iL).

**Figure 5:**
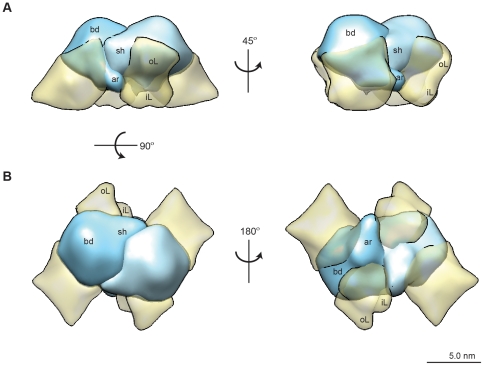
Arrangement of protein and lipid in the rHBsAg particle. (A) The arm (ar) and shoulder (sh) regions of each S-protein containing unit wrap tightly around the body (bd) of a neighboring unit to form a tight framework of protein around which lipids are interspersed. (B) The body (bd) organizes the outer lipid layer (oL), whereas the inner lipid (iL) layer surrounds the arm region of the S-protein containing densities that protrude into the inside of the VLP.

Extracting a single protrusion with associated lipid indicated how the trans-membrane S-protein-containing protrusions were positioned within the VLP lipid layer (Figure 4). The lipid layer itself was composed of two discrete regions, an outer section, likely composed of phospholipids [20], that was fairly uniform and ordered and whose ∼2.5 nm width corresponded to that of a lipid monolayer (Figure 4B, “oL” and Figure S8C), and an inner section, likely composed of non-polar triglycerides [20], that was more amorphous in appearance and more variable in width (Figure 4B, “iL” and Figure S8C). We segmented each “knuckle” of protein density into three general regions called body, shoulder, and arm (Figure 4B, “bd”, “sh”, “ar”), that were differentially positioned with respect to the ordered, outer lipid monolayer and the disordered, inner nonpolar lipid layer. The arm and shoulder regions of each S-protein containing density wrapped around the body region of a neighboring protein density to provide a solid framework of repeating S-protein densities around which lipids were organized [20], [21], [31], [35] (Figure 5A). Notably, the body of the repeating S-protein framework organized the outer lipid monolayer, in contrast to the inner lipid monolayer which was less organized and immersed the arm regions of the protein densities (Figure 5B).

### Quantitation of 3D Structural Results

In order to further define the structural properties of rHBsAg VLPs, we performed quantitative measurements of the cryoTEM map (Table 2 and Figure S8). Measurement of the size of the 3D map revealed diameters ranging 19.4–21.7 nm, with the twofold rotational axis view having an average diameter of ∼22 nm, the threefold rotational axis having an average diameter of ∼21 nm, and the fourfold rotational axis view having an average diameter of ∼20 nm (Figure S8A). Whereas previous studies interpreted the observation of multiple size populations in cryoTEM images as being physically different particle sizes [19], our analysis suggested that these are the same particle viewed from different rotational axes.

**Table 2:** A Summary of the Structural Properties of the yeast-derived rHBsAg VLP determined by CryoTEM.

	Property	Result	Literature References
**rHBsAg Particle**	Particle Symmetry	Octahedral	[19], This work
	Particle Symmetry Axes	4∶3∶2	[19], This work
	Particle Diameter, 3-fold view	21 nm	This work
	Particle Diameter, 4-fold view	20 nm	This work
	Particle Diameter, 2-fold view	22 nm	This work
	Number of Protrusions	24	[19], This work
	Protrusion Spacing, 2- and 3-fold	7 nm	This work
	Protrusion Spacing, 4-fold	9 nm	This work
	Protrusion Height	2 nm	[19], [21], This work
**rHBsAg Protein Monomers**	Number of Protein Monomers per Protrusion	4	[21], This work
	Number of Protein Monomers per Particle	96	[21], This work
	Number of Cysteine Residues per Protrusion	32	[21], This work
	Number of Cysteine Residues per Particle	768	[21], This work
**rHBsAg lipid and protein**	Width of Outer Lipid Monolayer	2.5 nm	This work
	Width of Inner Lipid Layer	Variable	This work
	MW of Protein Monomer, extracellular	4.5 kDa	This work
	MW of Protein Monomer, Outer Lipid monolayer	12.8 kDa	This work
	MW of Protein Monomer, Inner Lipid Layer	7.8 kDa	This work
	Lipid Composition	22%	[19], [21], This work
	Protein Composition	78%	[19], [21], This work

The S-protein containing protrusions were spaced ∼7.0 nm apart along the threefold rotational axis, ∼7.4 nm apart along the twofold rotational axis, and ∼9.2 nm apart along the fourfold rotational axis (Figure S8B) and projected ∼2.2 nm from the smooth surface (Figure S8C). The outer lipid layer measured ∼2.5 nm in width, and the inner lipid layer ranged ∼1.5–2.2 nm in width (Figure S8C). This is in agreement with a Dane particle diameter of 42 nm and a nucleocapsid (HbcAg) radius of 13.5 nm [20], [35], which leaves 6.5 nm for the rHBsAg protein-lipid layer, and removing 2.2 nm protrusions, leaves 4.3 nm total for the ordered outer lipid monolayer (∼2.5 nm) and the variable inner nonpolar lipid layer (∼2.2 nm).

Based on the total volume occupied by protein densities and lipid densities, the rHbsAg particle had a protein and lipid composition of 78% and 22%, respectively (Table S4). The volume occupied by individual protein densities corresponded to a MW of ∼100 kDa, which can accommodate four S-protein monomers of ∼25 kDa MW each [21], to give 96 S-protein monomers per rHBsAg VLP. This supports earlier work showing that mAbs RF1 and A1.2 only bind higher MW bands of S-protein in a non-denaturing Western Blot [30] and the dimer of dimers model [21] in which each protrusion consists of two S-protein dimers for a total of 48 dimers per rHBsAg VLP. Other results show that better-maintained protrusion heights and increased antigenicity are observed by in-solution AFM, SPR, and ELISA after rHBsAg VLP maturation or redox treatment [12], [14], [22] (Figures 1, S1, S4, S5). Moreover, the most common escape mutant of HBsAg in HBV alters the epitopes of the “a” determinant—an immunodominant region that comprises the cysteine- and proline-rich RF1 binding site [30], [31]. Together, our results and these studies suggested that these “knuckle”-like protrusions house clinically relevant epitopes of HBsAg VLPs.

The body of each protrusion was partially submerged in the lipid monolayer, with ∼18 kDa of tetrameric S-protein protruding into the extracellular space (Table S4), able to accommodate a stretch of amino acid residues (AA 105–156) in the antigenic loop most implicated in infectivity and antigenicity [27], [28]. The arm and shoulder wrapped around the body of a neighboring protein density and were partially submerged in the lipid monolayer, with ∼31 kDa total of tetrameric S-protein from both arm and shoulder regions protruding into the nonpolar lipid layer on the inside of the VLP. Together, the regions of the body, arm, and shoulder that reside in the ordered lipid monolayer accounted for ∼51 kDa of tetrameric S-protein density. These numbers were in agreement with those determined by existing structural studies and previous estimates based on primary sequence analysis [21], [31], though more of the S-protein tetramer (∼50%) appeared to be submerged in the lipid layer in our structure than what was previously observed. This is likely due to tighter packing resulting from our homogenous recombinant S-protein (versus S, M, L in other structures) and because we used a fully and uniformly matured particle via redox treatment for structure determination.

## Discussion

### Structural Model for rHBsAg VLP Antigenicity

Together with previously published results, the analysis of the 3D TEM structure presented here reveals a structural model for the rHBsAg vaccine particle that is consistent with previous results regarding the antigenic features, lipid-protein arrangement, and overall particle architecture [17], [19], [20], [21], [27], [28], [31]. The rHBsAg VLP architecture is driven by a framework of 24 repeating protein units, each composed of a tetramer of S-protein monomers, for a total of 96 S-protein molecules surrounded by a tightly packed monolayer of mostly polar lipids. Particle stability is conferred by trans-membrane helix-helix interactions between S-protein monomers and lipid-protein associations [20], [22], [35]. The in-membrane portion of S-protein is cemented in the lipid monolayer on the inside of the VLP by nonpolar lipids. The top of each S-protein tetramer likely comprises the “a” determinant and protrudes slightly into the extracellular space, where it presents the vaccine epitope. The main antigenic loops of a tetramer of S-protein monomers, observed as a protrusion in the 3D structure, consists of 32 cysteine residues that are vital for forming the virion surface like structure of this key antigenic protrusion with 36 prolines providing some conformational flexibility. The epitopes on these surface protrusions, relying on the proper disulfide bond pairings at the dimeric and tetrameric interface, are critically important for HBsAg VLP to elicit neutralizing titers against HBV. Therefore, it is important to assure the desired epitope formation, determinants for vaccine potency, and particle stability during bioprocess and subsequent formulation and storage.

### A Novel Toolbox for Vaccine Characterization

We have presented, using rHBsAg particle as a case study, two non-intrusive methods, cryoTEM and AFM, for structural characterization of recombinant VLPs. In addition, two antigenicity analysis methods using monoclonal antibodies, SPR and ELISA, provide quantitative tools to track the development of clinically relevant epitopes or to monitor changes during bioprocessing or storage and to ensure manufacturing consistency and product stability [12], [14]. These structural and functional methods are beneficial during early development phases of a vaccine candidate and post licensure to support routine manufacturing and process improvement because *in vitro* antigenicity, in an epitope-specific manner, is the best surrogate marker for *in vivo* immunogenicity or vaccine efficacy.

While the combined structural and functional vaccine characterization enabled by this toolbox has great utility, the methods presented here are not without potential limitations. The structural component of the toolbox requires sophisticated equipment and a high degree of user expertise to collect and analyze data. Moreover, while these tools are capable of providing quantitative data on particle size and morphology, they are not well suited to high-throughput applications. For high-throughput studies, typically conducted at the early stages of development, more standard technologies such as dynamic light scattering (DLS), high performance size exclusion chromatography (HP-SEC), and, more recently, nanoparticle-tracking analysis (NTA), are more practical. Nevertheless, the most complete understanding of a given vaccine drug product will likely be achieved by combining the high-throughput methods with the orthogonal tools presented here.

While these methods were demonstrated with recombinant protein based VLPs, these techniques should also be useful for characterizing other virion- or VLP-based vaccines or drugs for gene therapy based on live or attenuated viruses. Depending on the biologic in question, a few considerations are provided here to aid in the application of this toolbox. Heterogeneity in virion or VLP size, morphology, and composition could confound the ease with which 3D information is obtained. For VLPs derived from non-enveloped viruses (e.g. HPV, Norovirus, Rotavirus) this tends to be less of a concern, as these particles tend to be fairly homogenous in size, morphology, and composition. For vaccines derived from enveloped viruses (e.g. Respiratory Synctial Virus, Influenza, Herpes Virus, HIV, HBV), application of the toolbox may require additional care to overcome limitations imposed by heterogeneity in size, shape, and composition. In the case of the current work, structural information was more readily obtained after reducing population heterogeneity via recombinant technology, and since this approach is increasingly the trend in vaccine engineering, this type of application should become more accessible in the future.

A strong understanding of the structure-activity relationship is crucial for all the stages of vaccine design, development, and manufacturing. In the recent case of the successful production of a quadrivalent HPV vaccine (Gardasil® licensed in 2006), ∼15 years elapsed between the initial report of VLP formation with a single capsid protein and the appearance of the commercial product [1], [2]. We hope that the toolbox of epitope-preserving and contemporary methods presented here (Table 1) will facilitate the structural and functional characterization of other VLPs in the future, thus expediting the development process for vaccines under clinical trials and assuring manufacturing consistency for licensed vaccines.

## Materials and Methods

### Recombinant HBsAg VLPs

rHBsAg was over-expressed and purified from Saccharomyces cerevisae as previously described [36] and subjected to in-solution AFM, SPR, and solution competition ELISA before and after spontaneous (heat)- or redox-assisted maturation [14].

### SPR (Biacore) Antigenicity Assessment

Real time antigenicity monitoring and end point antigenicity assessment were performed as described recently [23]. Briefly, using a Biacore 3000 instrument, CM5 sensor chips were prepared by covalently coupling rabbit-anti-mouse IgG Fcγ antibodies (RAMFcγ) through the carboxylate groups in the dextran matrix on the sensor chip and the amine groups on the RAMFcγ using a Biacore Coupling Kit. The immobilized RAMFcγ antibodies were used to capture a Hepatitis B murine monoclonal antibody (mAb) IgG – RF1 or A1.2. VLP samples (10 µg/mL rHBsAg) were injected with a constant volume and constant flow rate, and extent of binding measured as the mass increase at the sensor chip. Results from the samples are compared to a reference lot tested in adjacent cycles.

### Fluorescence ELISA for IC50 Antigenicity Measurement

Briefly, a fixed amount of rHBsAg on plate and varying amount of rHBsAg VLPs in solution were allowed to compete for binding with a constant concentration of anti-HBsAg mAbs at 10 or 20 ng/mL. Serial dilutions (1.5-fold) of a reference lot and of test samples were performed on the VLP coated assay plate using a Beckmann Biomek® 2000 or 3000 Laboratory Automation Workstation. Bound antibody was quantitated with an alkaline phosphatase-conjugated rabbit anti-mouse IgG antibody and 4-methylumbelliferyl phosphate (4-MUP) (Virolabs, Chantilly, VA) as a substrate [37], [38]. IC50 values were calculated using a four parameter logistic fit with GraFit software or Excel, and compared to that of a reference lot tested on the same plate.

### In-solution AFM

Samples were analyzed by in-solution AFM as described previously [14]. Briefly, aqueous sample was deposited on a freshly cleaved mica surface (1/4″ diameter) at room temperature to allow passive adsorption, followed by a gentle rinse with de-ionized water. AFM images were collected with Digital Nanoscope III MultiMode AFM TappingModeTM (Digital Instrument, Santa Barbara, CA) in fluid at ambient temperature. The quantitative statistical assessment was performed with the image software supplied by Digital Instrument.

### CryoTEM imaging and analysis

#### CryoTEM sample preparation and imaging

The samples were preserved in a thin layer of vitrified ice over continuous Carbon layered over C-Flat holey carbon films supported on 400 mesh copper grids. Low dose cryoTEM was performed on a FEI Tecnai Spirit EM operating at 120 keV equipped with a FEI Eagle CCD camera. 218 images were acquired using a dose of ∼25 e-/Å2 at 52,000× (0.21 nm/pixel) magnification and a defocus range of ∼1–3 µm underfocus.

#### CryoTEM image processing and initial models

Particles were selected using image processing interface APPION [39] by 1) template matching [40], using filtered versions of projections of EMDB 1158 and 1159 [19] and 2) difference of Gaussians algorithm, “DoGpicker”, [41] to produce stacks of 1) 22,946 particles (4.24 A/pixel) and 2) 33,962 particles (2.12 A/pixel). The CTF of each image was estimated using ACE [42] and corrected using EMAN [43]. For 3D reconstruction, we utilized the following initial model approaches: a) The maps produced by Gilbert et al. [19] (http://www.ebi.ac.uk/msd/index.html, EBI # 1158, 1159) were low-pass filtered to 10 nm, and scaled to a sampling size of 0.212 nm/voxel; b) Initial models were generated using common lines in EMAN [43] with C4 symmetry.

#### CryoTEM 3D reconstruction: refinement

We used EMAN [43] and Spider [44] to produce a 3D map. Particles were subjected to 8 rounds of EMAN [43] refinement using 4 rounds at 5° angular increments followed by 4 rounds at 3° angular increments. Octahedral symmetry was imposed. After each refinement iteration, classes were subjected to reference-free classification using Spider [44] to eliminate subclasses that match poorly to the initial model. The final reconstruction analyzed in this manuscript utilized particles selected using template matching [40], the 1159 Gilbert map [19] as an initial model, and resulted in a 3D map with a nominal resolution of ∼1.5 nm according to the FSC0.5 resolution criterion. A total of 20,335 particles passed selection criteria allowing them to contribute to the final 3D map.

#### Segmentation of 3D volume obtained by cryoTEM

The resulting 3D map was segmented using the “Segger” and “Mask” functionalities in the visualization package chimera [45]. Protein and lipid densities were determined via visual assessment of the resulting “Segger” regions and examination of the 3D map at varying density thresholds. In order to physically segment the protein and lipid densities from the 3D volume, the “Mask” command was utilized. Segmentation of individual lipid layers and the individual protein densities were also achieved using “Segger”.

#### Volume and Molecular Weight calculations of segmented 3D map

The volume of each segmented unit was obtained using the “Measure Volume” functionality in chimera [45]. Briefly, the volume measured for a single segmented region of protein density was 141.4×10^3^ Å^3^ at a display threshold of 2.8, giving a MW of 117.6 kDa [46], corresponding to 4 S-protein monomers. The volume corresponding to this unit was normalized to 100 kDa for further calculations. The volume of protein residing in different parts of the VLP was obtained by the “Measure Volume” functionality after using the “Eraser” sphere in chimera.

## Supporting Information

Figure S1 **HBsAg Antigenicity for different mAbs.** (A) Solution competition ELISA profiles for deriving IC50 vales and the fitted lines for RF1 on HBsAg samples with different degrees of maturation. (B) Different mAbs displayed different level of sensitivity for HBsAg products with varying end point antigenicity.
(EPS)

Figure S2 **HBsAG particle size distributions.** (A) AFM images obtained in solution and statistical analysis for size distribution determination on two independent production batches. (B) Quantitative particle size distribution analysis of 266 particles extracted from CryoTEM images revealed a maximum Feret's diameter of 20.6+/−2.1 nm and a minimum Feret's diameter of 15.7+/−1.7 nm. The circularity of particles was determined by taking the ratio of maximum and minimum Feret's diameters, and measured 0.89+/−0.03 for HbSAg particles; a circularity measure of >0.92 is considered spherical.
(EPS)

Figure S3 **AFM images obtained in solution for different processes of HBsAg VLPs prior to and post KSCN treatment.** More surface features and less flattening were observed after the oxidative KSCN treatment.
(EPS)

Figure S4 **CryoTEM images of rHBsAG particles (A) before and (B) after DTT treatment, and (C) of plasma-derived HBsAg particles.** Particle boundaries were more defined and spikes were better preserved for rHBsAG particles that were not treated with DTT and that are plasma-derived.
(TIF)

Figure S5 **Class average analysis of rHbSAg particles from CryoTEM data.** (A) 2D alignment and classification of a subset of particles revealed (B) two size populations of particles with small (left two panels) and large (right two panels) diameters. (C) Automated determination of average particle diameters from class averages revealed a bimodial size distribution with mean values of 20.6+/−0.8 nm and 22.5+/−0.6 nm.
(EPS)

Figure S6 **A summary of the 3D maps generated during model refinement with octahedral symmetry.** (A) Using Gilbert initial model (1) 1158 and particles selected by template selection with 1158, (2) 1159 and particles selected by template selection with 1159, (3) 1158 and particles selected by DoG picker, and (4) 1159 and particles selected by DoG picker. (B) Using EMAN StartAny with C4 Symmetry initial models generated with (1) 1158 particle stack and refined with 1158 particle stack, (2) 1159 particle stack and refined with 1159 particle stack, (3) DoG particle stack and refined with 1158 particle stack, and (4) DoG particle stack and refined with 1159 particle stack.
(EPS)

Figure S7 The refined 3D map with octahedral symmetry with the front half cut away to reveal an internal particle that is empty. Corresponds to Figure 3B.
(EPS)

Figure S8 **Quantitative measurements of the 3D map.** (A) Particle diameters for each of the 2-fold, 3-fold, and 4-fold rotational axis views. The average particle diameter is 21.7 nm, 21.0 nm, and 20.3 nm respectively. (B) Measurement of the distance between protrusions reveals spacing of ∼7.4 nm along the 2-fold axis, ∼7.0 nm along the 3-fold axis, and ∼9.2 nm along the 4-fold axis. (C) Measurement of the dimensions of the lipid layer and the distance with which the protein protrudes from the VLP.
(EPS)

Table S1 Initial discovery of VLPs and key vaccines for human use or in clinical trials based on VLP approach (1968 to 2011).
(DOC)

Table S2 Quantitative analysis of RF1 epitope in HBsAg VLPs using competitive ELISA (rel IC50) for lot-to-lot consistency.
(DOC)

Table S3 Structural characterization with CryoTEM on subviral particles of HBV and the main conclusions.
(DOC)

Table S4 Quantitative analysis of Segmented CryoTEM Volume.
(DOC)
