# Supplementary figures and images for: Toolbox for Non-Intrusive Structural and Functional Analysis of Recombinant VLP Based Vaccines: A Case Study with Hepatitis B Vaccine
Source: PLoS One. 2012 Apr 6;7(4):e33235. doi: 10.1371/journal.pone.0033235 (PMC3320896; doi:10.1371/journal.pone.0033235)

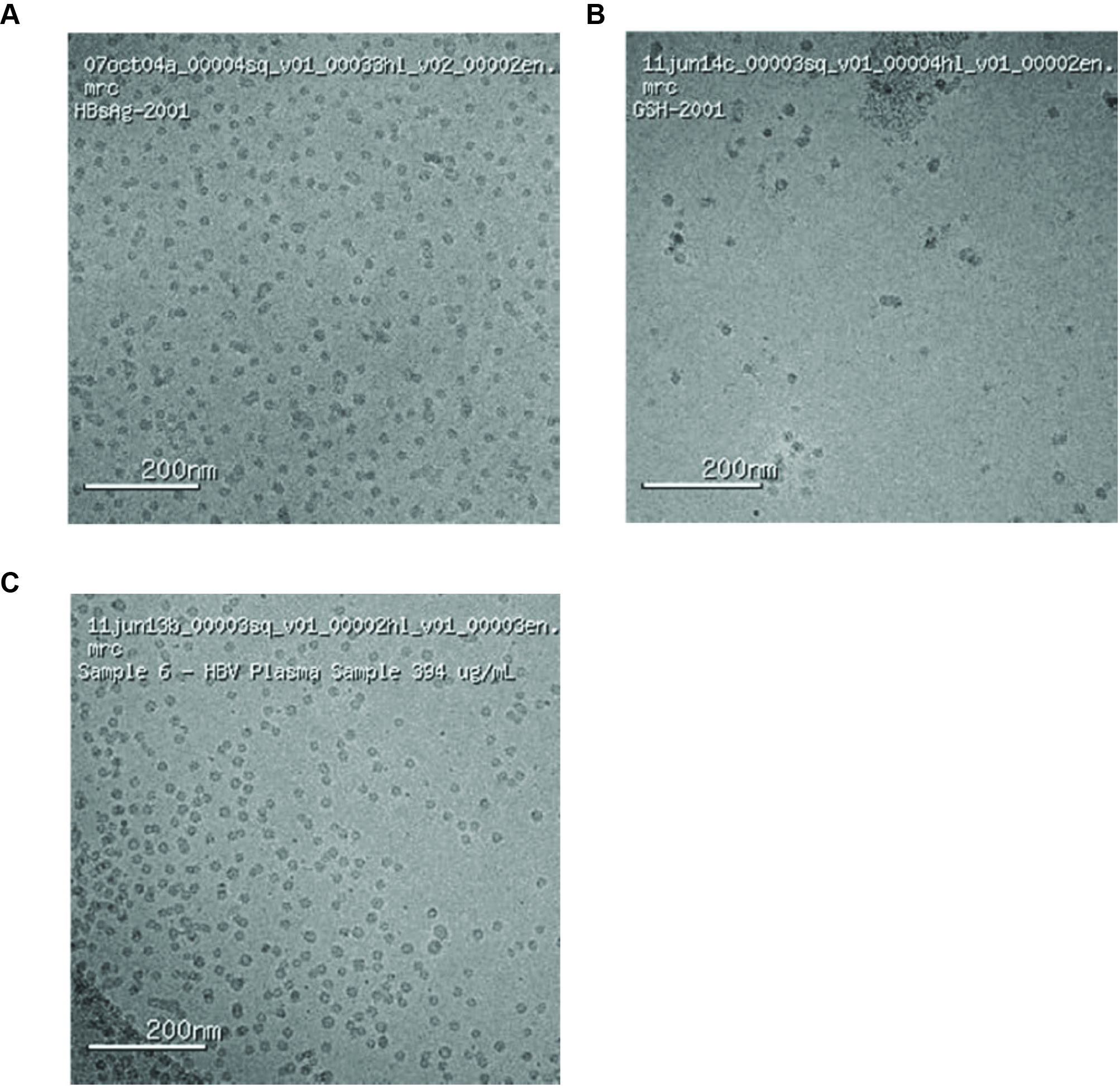

Supplement: Figure S4 — CryoTEM images of rHBsAG particles (A) before and (B) after DTT treatment, and (C) of plasma-derived HBsAg particles. Particle boundaries were more defined and spikes were better preserved for rHBsAG particles that were not treated with DTT and that are plasma-derived. (TIF) [file pone.0033235.s004.tif]
