# Supplementary material for: Toolbox for Non-Intrusive Structural and Functional Analysis of Recombinant VLP Based Vaccines: A Case Study with Hepatitis B Vaccine
Source: PLoS One. 2012 Apr 6;7(4):e33235. doi: 10.1371/journal.pone.0033235 (PMC3320896; doi:10.1371/journal.pone.0033235)
Supplement: Table S1 — Initial discovery of VLPs and key vaccines for human use or in clinical trials based on VLP approach (1968 to 2011). (DOC) [file pone.0033235.s009.doc]

Table S1. Initial discovery of VLPs and key vaccines for human use or in clinical trials based on VLP approach (1968 to 2011).

|  | **Disease-causing Virus** | **Protein** | **Source** | **Year of Licensure** | **Trade Name** | **Company** |
| --- | --- | --- | --- | --- | --- | --- |
| **Initial Discovery** | Hepatitis B Virus | HBsAg (L, M, S) | Human plasma | 1968 | Australian Antigen |  |
| **Licensed Vaccines** | Hepatitis B Virus | HBsAg (L, M, S) | Human plasma | 1981 | Heptavax B ® | Merck |
|  | Hepatitis B Virus | HBsAg (S) | Yeast (s. cerevisiae) | 1986 | Recombivax HB ® | Merck |
|  | Human Papilloma Virus (Types 6,11,16,18) | L1 | Yeast (s. cerevisiae) | 2006 | Gardasil ® | Merck |
|  | Hepatitis E Virus | ORF2 | E. Coli | 2010 | Post-Phase III |  |
| **Clinical Trial Stage** | Influenza | HA, NA, M1 | Baculovirus | NA | Phase II | Novavax |
|  | Norovirus |  |  |  | Phase I | Ligocyte |
