# Supplementary material for: Toolbox for Non-Intrusive Structural and Functional Analysis of Recombinant VLP Based Vaccines: A Case Study with Hepatitis B Vaccine
Source: PLoS One. 2012 Apr 6;7(4):e33235. doi: 10.1371/journal.pone.0033235 (PMC3320896; doi:10.1371/journal.pone.0033235)
Supplement: Table S2 — Quantitative analysis of RF1 epitope in HBsAg VLPs using competitive ELISA (rel IC50) for lot-to-lot consistency. (DOC) [file pone.0033235.s010.doc]

Table S2. Quantitative analysis of RF1 epitope in HBsAg VLPs using competitive ELISA (rel IC50) for lot-to-lot consistency.

| **Sample Type** | **IC50 (ng/mL)** | **Relative IC50 (rIC50)** | **RSD % (n=3)** |
| --- | --- | --- | --- |
| HBsAg in solution-Reference | 426 | 1.00 | 7 |
| HBsAg in solution -Lot 1 | 776 | 0.55 | 7 |
| HBsAg in solution -Lot 2 | 758 | 0.56 | 2 |
| HBsAg in solution -Lot 3 | 668 | 0.64 | 12 |
| HBsAg in solution -Lot 4 | 750 | 0.57 | 8 |
| HBsAg on Adjuvant -Reference | 723 | 1.00 | 13 |
| HBsAg on Adjuvant -Lot 1 | 1381 | 0.53 | 11 |
| HBsAg on Adjuvant -Lot 2 | 1450 | 0.50 | 7 |
| HBsAg on Adjuvant -Lot 3 | 1100 | 0.66 | 13 |
