# Supplementary material for: Toolbox for Non-Intrusive Structural and Functional Analysis of Recombinant VLP Based Vaccines: A Case Study with Hepatitis B Vaccine
Source: PLoS One. 2012 Apr 6;7(4):e33235. doi: 10.1371/journal.pone.0033235 (PMC3320896; doi:10.1371/journal.pone.0033235)
Supplement: Table S3 — Structural characterization with CryoTEM on subviral particles of HBV and the main conclusions. (DOC) [file pone.0033235.s011.doc]

Table S3. Structural characterization with CryoTEM on subviral particles of HBV and the main conclusions. Here listed are the three different reports on 3D structure for plasma-derived or recombinant HBsAg VLPs.

| **HBsAg^a^** | **Source and Purpose** | **Size and Form** | **Key Conclusions** | **Literature Reference** |
| --- | --- | --- | --- | --- |
| Recombinant HBsAg (S) | Yeast (*s. cerevisiae*); Human Vaccine | ~22 nm; spherical | Octahedral Symmetry, 48 Dimers | This work |
| Naturally Occurring HBsAg (L, M, S) | Human Plasma; Basic Research | ~25 nm; tubular | Each protrusion contains a dimer of dimers | Short et. al (2009)^b^ |
| *In vivo* produced Recombinant HBsAg (S) | *In vivo* transgenic mice; Basic Research | ~ 22 nm; spherical | Octahedral Symmetry, Isometric | Gilbert et. al (2005)^c^ |

^a^Different forms of HBsAg have different number of amino acids: L, 400 or 389 aa; M, 281 aa; S, 226 aa. All three forms exist for plasma-derived HBsAg, whereas S form of HBsAg is the active component in the marketed HBV vaccines.

^b^Short JM, Chen S, Roseman AM, Butler PJ, Crowther RA (2009) Structure of hepatitis B surface antigen from subviral tubes determined by electron cryomicroscopy. J Mol Biol 390: 135-141.

^c^Gilbert RJ, Beales L, Blond D, Simon MN, Lin BY, et al. (2005) Hepatitis B small surface antigen particles are octahedral. Proc Natl Acad Sci U S A 102: 14783-14788.
