# Supplementary material for: Toolbox for Non-Intrusive Structural and Functional Analysis of Recombinant VLP Based Vaccines: A Case Study with Hepatitis B Vaccine
Source: PLoS One. 2012 Apr 6;7(4):e33235. doi: 10.1371/journal.pone.0033235 (PMC3320896; doi:10.1371/journal.pone.0033235)
Supplement: Table S4 — Quantitative analysis of Segmented CryoTEM Volume. (DOC) [file pone.0033235.s012.doc]

Table S4. Quantitative analysis of Segmented CryoTEM Volume^a^.

^a^ For details of the manner in which segmentation was done, see Figures S9 and S13.

|  | **Measured Volume (nm^3^)** |  | **Calculated Volume (nm^3^)** | **% Composition** |
| --- | --- | --- | --- | --- |
| Single Protein Protrusion | 1.41 x 10^2^ | All Protein, HbSAg VLP | 3.38 x 10^3^ | 77.38 |
| Single Lipid, 4-fold | 8.81 x 10^1^ | All Lipid, HbSAg VLP | 9.89 x 10^2^ | 22.62 |
| Single Lipid, 3-fold | 5.76 x 10^1^ |  |  |  |
|  | **Measured Volume (nm^3^)** | **kDa^a^** | **kDa, normalized^b^** |  |
| Single Protein Protrusion | 1.41 x 10^2^ | 117 | 100.28 |  |
| Single Protein Protrusion, Outside VLP | 1.41 x 10^2^ | 19.2 | 16.48 |  |
| Single Protein Protrusion, Outside VLP and Outer Membrane Layer | 1.41 x 10^2^ | 80.8 | 69.37 |  |
|  | **kDa** | **kDa per S-protein monomer** |  |  |
| Single Protein Protrusion, Outside VLP | 16.48 | 4.12 |  |  |
| Single Protein Protrusion, Outer Lipid Layer | 52.89 | 13.22 |  |  |
| Single Protein Protrusion, Inner Lipid Layer | 30.91 | 7.73 |  |  |

^b^Volume was converted to kDa using 1.21nm^3^/kDa as detailed in Harpaz Y, Gerstein M, Chothia C (1994) Volume changes on protein folding. Structure 2: 641-649.

^c^The volume corresponding to a single S-protein containing protrusion was set to 100kDa, and all other volume measurements were normalized to this value.
